# ARE COMPLETING ONLINE ASSIGNMENTS CORRELATED TO KIRKPATRICK’S LEVEL 2 AND 3 LEARNING OUTCOMES?

**DOI:** 10.1093/geroni/igad104.2273

**Published:** 2023-12-21

**Authors:** Huai Cheng

**Affiliations:** University of Pittsburgh, Pittsburgh, Pennsylvania, United States

## Abstract

**Background:**

Pre-class assignments such as online assignments are critical to a flipped classroom-based curriculum. However, it is unknown whether medical students are following online assignments and whether such compliance is associated with learning outcomes. The aim of this study was 1) to examine the compliance of the online assignments and 2) explore whether such compliance was associated with Kirkpatrick’s Level 2 and 3 learning outcomes. Summary of Works: Prior to classroom teaching, fourth-year medical students (MS4) were recommended (not required) to complete six online assignments. A simulated patient (SP) with chief complaint of fall was developed to assess students’ skills in interview, physical examination, and communication (Kirkpatrick’s level 2 learning outcome). Students’ clinical practice behavior was based on chart review (Kirkpatrick’s level 3 learning outcome). Pearson/Fisher correlations were used to examine the association of completing online assignments with SP performance and clinical practice behavior. Summary of

**Results:**

158 from Class 2017 participated in this study. The complete rate of 1-6 online assignment was 88%, 86%, 82%, 61%, 51%, 34%, respectively. Completing three of six online assignments was significantly associated with higher SP performance, but not clinical practice behavior. Discussion and

**Conclusions:**

Majority of MS4 didn’t complete online assignments. Online assignments should be required. Completing online assignments was associated with improving Kirkpatrick’s level 2, but not 3 learning outcomes. Take-home Messages: Online assignments need to be required. Improving Kirkpatrick’s level 2 learning outcome via a flip-based curriculum for MS4 might not improve Kirkpatrick’s level 3 learning outcomes.

